# Silicon and salicylic acid confer high-pH stress tolerance in tomato seedlings

**DOI:** 10.1038/s41598-019-55651-4

**Published:** 2019-12-24

**Authors:** Adil Khan, Muhammad Kamran, Muhammad Imran, Ahmed Al-Harrasi, Ahmed Al-Rawahi, Issa Al-Amri, In-Jung Lee, Abdul Latif Khan

**Affiliations:** 1 Natural and Medical Sciences Research Center, University of Nizwa, Nizwa, 616 Oman; 2 Plant Transport and Signalling Lab, School of Agriculture, Food and Wine, University of Adelaide, Glen Osmond, SA 5064 Australia; 3 School of Biological Sciences, Seoul National University, Seoul, Republic of Korea; 4 School of Applied Biosciences, Kyungpook National University, Daegu, Republic of Korea

**Keywords:** Physiology, Biogeochemistry, Ecology, Environmental sciences

## Abstract

Alkalinity is a known threat to crop plant growth and production, yet the role of exogenous silicon (Si) and salicylic acid (SA) application has been largely unexplored. Here, we sought to understand the beneficial impacts of Si and SA on tomato seedlings during high-pH (9.0) stress. Results showed that Si- and SA-treated plants displayed higher biomass, chlorophyll contents, relative leaf water and better root system than none-treated plants under alkaline conditions. Both Si and SA counteracted the alkaline stress-induced oxidative damage by lowering the accumulation of reactive oxygen species and lipid peroxidation. The major antioxidant defence enzyme activities were largely stimulated by Si and SA, and these treatments caused significantly increased K^+^ and lowered Na^+^ concentrations in shoot and root under stress. Moreover, Si and SA treatments modulated endogenous SA levels and dramatically decreased abscisic acid levels in both shoot and root. Additionally, key genes involved in Si uptake, SA biosynthesis, the antioxidant defence system and rhizosphere acidification were up-regulated in Si and SA treatments under alkaline conditions. These results demonstrate that Si and SA play critical roles in improving alkaline stress tolerance in tomato seedlings, by modifying the endogenous Na^+^ and K^+^ contents, regulating oxidative damage and key genes and modulating endogenous hormone levels. These findings will help to broaden our understanding regarding the physiological and molecular mechanisms associated with the alkaline soil tolerance in plants.

## Introduction

The widespread distribution of alkaline soils is a major limiting factor for agricultural productivity worldwide. According to one estimate, up to 831 × 10^6^ ha of the earth’s land is saline, of that 434 × 10^6^ ha is affected by soil alkalinity in more than 100 countries, causing severe damage to crop growth and loss of agricultural productivity^1,2^. Alkaline stress markedly hinders plant growth compared with salinity stress^3^. Despite this, the tolerance mechanisms of plants in response to alkaline stress have received less attention than the adaptive mechanisms of salinity stress^4–7^.

Under alkaline conditions, high rhizosphere pH is a significant factor affecting plant growth and development. Recent studies described the mechanism of alkaline soil tolerance, focusing on the ability of plants to acidify the rhizosphere *via* plasma membrane H^+^-ATPase-mediated proton secretion^2,8–11^. Several factors are known to regulate the activity of H^+^-ATPase. For example, *DNAJ HOMOLOG3* (J3) and *PROTEIN KINASE5* (PKS5) play critical roles in proton secretion by regulating the interaction between 14-3-3 proteins and the plant plasma membrane H^+^-ATPase^8,9^. Another study reported that *PIN-FORMED*2 (PIN2, an auxin efflux transporter) is required to tolerate alkaline stress conditions by regulating proton efflux in roots^10^. However, other adaptive mechanisms by that plants can tolerate alkaline stress have not yet been fully explored. This study aims to explore the alternative tolerance mechanisms, providing valuable knowledge for modern agriculture that can be used by breeders to select more productive crops.

Soils with alkaline salts enhance toxicity by producing reactive oxygen species (ROS), particularly, hydrogen peroxide (H_2_O_2_), superoxide (•O_2_^−^) and hydroxyl radicals (•OH)^12,13^. ROS production in plants may cause DNA injury and severe oxidative damage to cell organelles, lipids and proteins^14–17^. To cope with alkaline stress-induced oxidative damage, plant cells activate the endogenous antioxidant defence system, that includes enzymatic antioxidants, such as ascorbate peroxidase (APX), catalase (CAT), peroxidase (POD), polyphenol oxidase (PPO), superoxide dismutase (SOD), and non-enzymatic antioxidant metabolites^18–21^. SOD serves as the frontline defence and eliminates •O_2_^−^. This process also results in the formation of H_2_O_2_, that is detoxified by CAT and POD^22^. PPO and POD are oxidisers that catalyse lignin formation and other oxidising phenols^23^. APX enzymes use ascorbate to donate an electron and catalyse the transformation of H_2_O_2_ into H_2_O^24^. In plants, exogenously applied silicon (Si) enhances the ROS-scavenging ability by stimulating the antioxidant enzymes (APX, CAT, POD, PPO, SOD)^25–29^.

Plant perceive the environmental stimuli and trigger metabolic flux to activate a range of defence mechanisms. Many molecules have been suggested as signal messengers or transducers, such as SA, jasmonic acid, ethylene and calcium^30^. Salicylic acid (SA) is a natural molecule that plays a crucial role in regulating several physiological processes and plant resistance to environmental stresses^31–33^. It has been shown that SA provides resistance against low-temperature stress^34,35^, pathogen attack^36^, osmotic and salinity stress^37^, drought^38^, ozone^39^, herbicide^40^, heavy metals^33,41–43^ and, in addition, induces thermotolerance^44^. Furthermore, SA regulates the production of ROS, for example, •O_2_^–^, that is detoxified to H_2_O_2_ by SOD activation^31^. In addition to SA, Si has also been implicated in conferring similar beneficial impacts on plant growth physiology during stress conditions.

After oxygen, Si is the second most abundant element in the earth’s crust and has frequently been reported as a beneficial mineral element for plant growth and development^45,46^. Due to its strong affinity for other ions, Si is commonly found as silicic acid (H_4_SiO_4_), silicate (xM1_2_OySiO_2_) and as silica (SiO_2_)^47^. Si increases plant resistance to different biotic and biotic stresses^48–50^, such as salt and drought^51,52^, extreme temperature^53^, nutrient deficiency^54^, aluminium toxicity^55–58^, disease^54,59^ and damage by wild rabbits^60^. During stress, Si enhances multiple adaptive responses, for instance, hormonal regulation, the activation of antioxidant activities, the uptake of minerals and organic acid anions and the exudation of phenolic compounds^47,55,61–68^. To date, the interactive effects of SA and Si on the physiological, biochemical and molecular response of plants under alkaline stress have not been reported and warrants an in-depth investigation. To understand this, the current study selected tomato, one of the world’s largest fruit-bearing crops, with an annual production of over 150 × 10^6^ t, and a model for vegetative and developmental studies. We aimed to investigate an alternative mechanism of alkaline soil tolerance in plants. Our objective was to explore the effects of Si and SA on the plant performance under alkaline-stressed conditions and to dissect the Si- and SA-induced physiological, biochemical and molecular mechanisms responsible for alkaline soil tolerance in tomato plants.

## Materials and Methods

### Plant growth and treatment conditions

Seeds of tomato (*Solanum lycopersicum* L., LeaderF1; procured from Wadi Al-Lawami International) were surface-sterilised and rinsed with distilled water (dH_2_O) five times, followed by soaking in dH_2_O for 72 h. Thereafter, uniformly germinated seeds were transplanted to a seed tray containing sphagnum peat moss (moisture content 38.5%, pH 4.5–5.5, electrical conductivity 2.0 dS m^–1^, bulk density 0.7–1.0 mg m^–3^, grain size 125–250 μm and 91.1% organic matter: N, 800–2,500 mg kg^–1^; P, 150–850 mg kg^–1^; Na, 340 mg kg^–1^; NaCl, 850 mg kg^–1^) and placed in a greenhouse. Three-week-old plants were then transferred to pots (10 × 9 cm) filled with 200 g of peat moss, and dH_2_O (50 mL per pot) was applied to all seedlings for acclimatisation to the conditions. The experiment was conducted in the form of a randomised complete block design with three replications in a net plot size of 10 × 9 cm. For stress treatment, one set of tomato seedling was supplied with pH 6.0 buffer (adjusted using HCl), along with Si (Na_2_SiO_3_.5H_2_O), SA and combined Si + SA, respectively. Another set of plants was treated with pH 9.0 buffer (adjusted using NaOH), along with Si, SA and Si + SA, respectively. To stimulate alkaline stress, we used NaHCO_3_ (1 mM) and Na_2_CO_3_ (80 µM), in pH 9.0 buffers^28,69,70^. We used 1 mM Si because Kim *et al*.^28^ showed it improved rice plant growth and resistance against wounding and flooding stress. Similarly, 100 µM SA was used because it extended tolerance to plant growth. After 5 weeks, significant differences were observed in the shoot and root growth attributes among the control, Si-, SA- and Si + SA-treated plants, at each pH. The SA concentrations were selected based on our preliminary tests. Each pot was supplied with 50 mL buffer every alternate day, for 5 weeks. After 5 weeks, tomato plants were harvested. Each treatment consisted of 21 plants, and the experiment was repeated three times. For each treatment, nine tomato seedlings were randomly selected from the 21 seedlings, with three replications. Shoot and root parameters were determined, followed by the quantification of fresh mass (FM) and dry mass (DM) of both root and shoot.

### Chlorophyll *a* (Chl *a*) and chlorophyll *b* (Chl *b*) and leaf relative water content (LRWC) quantification

Tomato leaves (200 mg) were ground with 80% acetone for the extraction of photosynthetic pigments (Chl *a*, Chl *b* and carotenoid). Chl *a* and Chl *b* were estimated, as described by Sumanta *et al*.^71^, with absorbance at 663 nm. The absorbance of the carotenoid assay was recorded at 645 nm.

The relative water content (RWC) was estimated, as described by Abdel Latef and Tran^25^. The second leaves were excised, and their fresh mass (FM) was determined immediately. After floated on deionised water for 5 h, the saturated mass (SM) was recorded. Then, the leaves were dried to constant weight at 80 °C to measure their dry mass (DM). The RWC was calculated as follows: RWC (%) = D [(FM – DM)/ (SM – DM)] × 100. The experiment was repeated three times, with three replications each.

### Determination of lipid peroxidation and •O_2_^–^ during stress conditions

The extent of lipid peroxidation was determined by measuring the malondialdehyde (MDA) content based on the method of Ohkawa *et al*.^72^ For this assay, the tissue homogenate was extracted using phosphate buffer (10 mM, pH 7.0). The reaction mixture consisted of acetic acid (1.5 mL of 20%, pH 3.5), sodium dodecyl sulphate (SDS; 0.2 mL of 8.1%), thiobarbituric acid (1.5 mL of 0.81%) and tissue homogenate (0.2 mL). After heating the reaction tube for 60 min, it was placed at room temperature for 15 min, followed by the addition of 5 mL butanol:pyridine (15:1, v/v) solution. The optical density of the upper organic layer (pink solution) was recorded at 532 nm. 1,1,3,3-Tetramethoxypropane was considered as an external standard, and the experiment was repeated three times.

The rate of •O_2_^–^ generation was measured by the method of Gajewska and Skłodowska^73^. Fresh plant powder (1 g) was immersed in phosphate buffer (pH 7.0), containing sodium phosphate (10 mM), nitroblue tetrazolium (0.05%, w/v) and sodium azide (10 mM). The mixture was maintained at room temperature for 1 h, and then 5 mL was transferred to an empty test-tube and heated at 85 °C for 15 min in a water bath, cooled on ice and vacuum-filtered. The spectrophotometric absorbance of the sample was read at 580 nm. The experiment was replicated three times.

### Determination of antioxidant enzymatic activity

Protein was extracted by grinding leaf sample (100 mg) with potassium phosphate buffer (100 mM, pH 6.8) containing ethylenediaminetetraacetic acid (0.2 mM EDTA). After centrifugation (12,000 × *g* for 30 min), the supernatant was transferred to an empty tube and the total protein quantified based on the Bradford^74^ protocol. The experiment was replicated three times.

The established protocol of Kar and Mishra^75^ with slight modification was used to determine the activity of the antioxidant enzymes APX, CAT, POD and PPO. In brief, leaf sample (100 mg) was ground using a chilled mortar and pestle, then combined with phosphate buffer (0.1 M, pH 7.0) to homogeneity, followed by centrifugation (10,000 rpm for 30 min) in a refrigerated centrifuge. To quantify POD, crude extract (100 μL) was combined with potassium phosphate buffer (0.1 M, pH 6.8), H_2_O_2_ (50 μL of 50 µM) and pyrogallol (50 μL of 50 µM) and incubated at room temperature for 5 min, followed by the addition of H_2_SO_4_ (5% v/v). The extent of purpurogallin production was measured by the optical density at 420 nm. The same wavelength of measurement (420 nm) and a similar reaction mixture composition to POD, but with H_2_O_2_ (50 µM), were used to quantify the PPO. The CAT activity was assayed, as described by Aebi^76^. Briefly, the crude enzyme extract was added to H_2_O_2_ (0.2 M) in phosphate buffer (10 mM, pH 7.0) and the CAT activity was determined as the decrease in absorbance at 240 nm and expressed as units (1 U of CAT was defined as μg H_2_O_2_ released mg protein^–1^ min^–1^). To assay APX activity, the samples were first extracted with 1 mL of phosphate buffer (50 mM, pH 7.0) containing ascorbic acid (1 mM) and EDTA (1 mM), then homogenised (50 Hz for 30 s), and the homogenate centrifuged (4,830 × *g*, 4 °C for 15 min). The supernatant was mixed with a phosphate buffer solution (pH 7.0) containing ascorbic acid (15 mM) and H_2_O_2_ (0.3 mM). The reaction mixture was analysed at 290 nm. One unit of APX was defined as the variable quantity of absorbance at 290 nm min^–1^. All the enzymatic assays were repeated three times, with three replications each.

### Scanning electron microscopy (SEM) of root samples

For SEM, the method reported by Al-Harrasi *et al*.^77^ and Peckys *et al*.^78^ was used. The root part was selected from the same portion of root from all the treatments. Immediately, the sample was fixed in Karnovsky solution (2.5% glutaraldehyde in sodium cacodylate buffer and glutaraldehyde), followed by secondary fixation through the addition of osmium tetroxide (OsO_4_) at 4 °C for 1 h. After the OsO_4_ was decanted, the samples were washed twice with dH_2_O. Samples were successively dehydrated using a series of increasing ethanol concentrations (30%, 50%, 70%, 90% and 100%, three times each). After dehydration, samples were dried using a critical point dryer. The dried samples were mounted on an aluminium stub (10 × 10 mm) and coated with plutonium for 3 min. Samples were visualised individually under a scanning electron microscope.

### Endogenous Si quantification by inductively coupled plasma–mass spectrometry (ICP–MS)

Si was quantified in 0.05 g of ground, freeze-dried tomato root and leaves, by following the method described by Bilal *et al*.^79^, using an ICP-MS instrument (Optima 7900DV, Perkin-Elmer, USA). The experiment was repeated three times, with three replications each.

### RNA extraction, cDNA synthesis and quantitative real-time PCR (qPCR)

RNA was extracted from tomato shoots and roots by using the extraction buffer (0.25 M NaCl, 0.05 M Tris–HCl pH 7.5, 20 mM, EDTA, 1% w/v SDS, 4% w/v polyvinyl pyrrolidone), as described by Liu *et al*.^80^ Before the addition of sample, 750 μL of the extraction buffer and chloroform:isoamyl alcohol (CI; 24:1 v/v) were placed in a 2-mL RNase-free microcentrifuge tube, followed by the addition of β-mercaptoethanol (40 μL). Fine powder (100 mg) of the sample was carefully transferred to the 2-mL tube. The mixture was vortexed, incubated (20 °C for 10 min) and centrifuged (12,000 × *g*, 4 °C for 10 min). Around 600 μL of the supernatant was transferred to a separate 2-mL tube. The same volume of CI was added to all the tubes. The solutions were mixed gently and centrifuged (12,000 × *g*, 4 °C for 10 min). The upper layer was transferred to an empty 1.5-mL microcentrifuge tube, and 1/10 volume of sodium acetate (3 M, pH 5.2) was added. For precipitation, absolute ethanol (two volumes) was added. After gently mixing, the tubes were incubated (4 °C for 45 min) and then centrifuged (12,000 × *g*, 4 °C for 10 min). The pellet was dissolved in DEPC-treated water (200 μL), and lithium chloride (500 μL of 10 M) was added to the solution. The solutions were mixed gently and placed on ice for 60 min. In the final step, the samples were centrifuged (12,000 × *g*, 4 °C for 10 min), and the pellet was washed with 70% ethanol. After removing the ethanol, the pellet was air-dried and then dissolved in DEPC-treated water (50 μL). The RNA quality was checked on agarose gel electrophoresis and quantified using the Qubit 3.0 RNA broad-range kit.

The extracted RNA (>100 ng/µL) was used for cDNA synthesis. Master Mix was prepared using 25X dNTPs, the reverse transcriptase enzyme, random primers and nuclease-free water. RNA was added to Master Mix, according to its concentration (e.g., for each 100 ng/µL RNA, 10 µL was taken for the cDNA synthesis). PCR was performed in a thermocycler under specific conditions (25 °C for 10 min, 37 °C for 2 h and 85 °C for 5 min). The synthesised cDNA was refrigerated at –80 °C until further use.

The synthesised cDNA was used for the amplification of genes (Supplementary Table S1**)**. A total of 12 genes related to the Si and SA biosynthesis pathways and antioxidant enzymes were analysed in each sample. *Actin* was used as the reference gene for all the primers. Power SYBR Green Master Mix was used for the thermocycler PCR reaction, and primers (forward and reverse, 10 pM) were used for all the 12 genes. For each sample, the reaction was performed in triplicate to minimise errors and contaminations. The initial reaction step at 94 °C for 10 min was followed by 35 cycles at 94 °C for 45 s, 65 °C for 45 s and 72 °C for 1 min, and, finally, the extension step occurred at 72 °C for 10 min. The threshold level of 0.1 was set for gene amplification. The experiment was repeated three times, with three replications each.

### SA extraction and quantification

SA was extracted and quantified from freeze-dried tomato sample, based on Seskar *et al*.^81^, as described by Shahzad *et al*.^82^ The extracted samples were analysed by high-performance liquid chromatography (HPLC) using a Shimadzu device outfitted with a fluorescence detector (Shimadzu RF-10AxL) and a C18 reverse-phase HPLC column (HP Hypersil ODS, particle size 5 μm, pore size 120 Å; Waters). The excitation and emission wavelengths were 305 and 365 nm, respectively. The flow rate was set at 1.0 mL min^–1^ **(**Supplementary Table S2**)**. The experiment was repeated three times, with three replications each.

### Abscisic acid (ABA) extraction and quantification

Endogenous ABA was extracted and quantified, as described in Bilal *et al*.^79^ and Shahzad *et al*.^83^ Briefly, the extracted samples from ground freeze-dried plants were supplemented with [(±)-3,5,5,7,7,7-d6]–ABA as the internal standard and then analysed by gas chromatography–mass spectrometry (6890 N network GC system, and 5973 network mass selective detector; Agilent Technologies, Palo Alto, CA, USA). The method spectra were recorded in selected ion mode at *m/z* 162 and 190 for Me–ABA and *m/z* 166 and 194 for Me–[2H6]–ABA. The ABA was calculated by comparing the endogenous peak with the respective standards. The experiment was repeated three times, with three replications each **(**Supplementary Table S2**)**.

### Statistical analysis

All graphs and data analyses were performed using GraphPad Prism (v7.02; San Diego, CA USA). All data are shown as mean ± SE. A repeated measures ANOVA based on general linear model was performed using multiple comparison among treatments via Tukey test. The significance between each treatments at a given pH was shown *(*P* < 0.05),**(*P* < 0.01), and ***(*P* < 0.001) and “ns” indicates non-significant differences. Additionally, a Duncan multiple range test (SAS v9.0, NY USA) was also performed for all the treatment to reveal significant to non-significant treatments by maintain (*P* < 0.05) and representing by different alphabets.

## Results

### Comparison of tomato shoot and root growth at the control and alkaline pH, and the interaction of Si and SA applications

**Figure 1:**
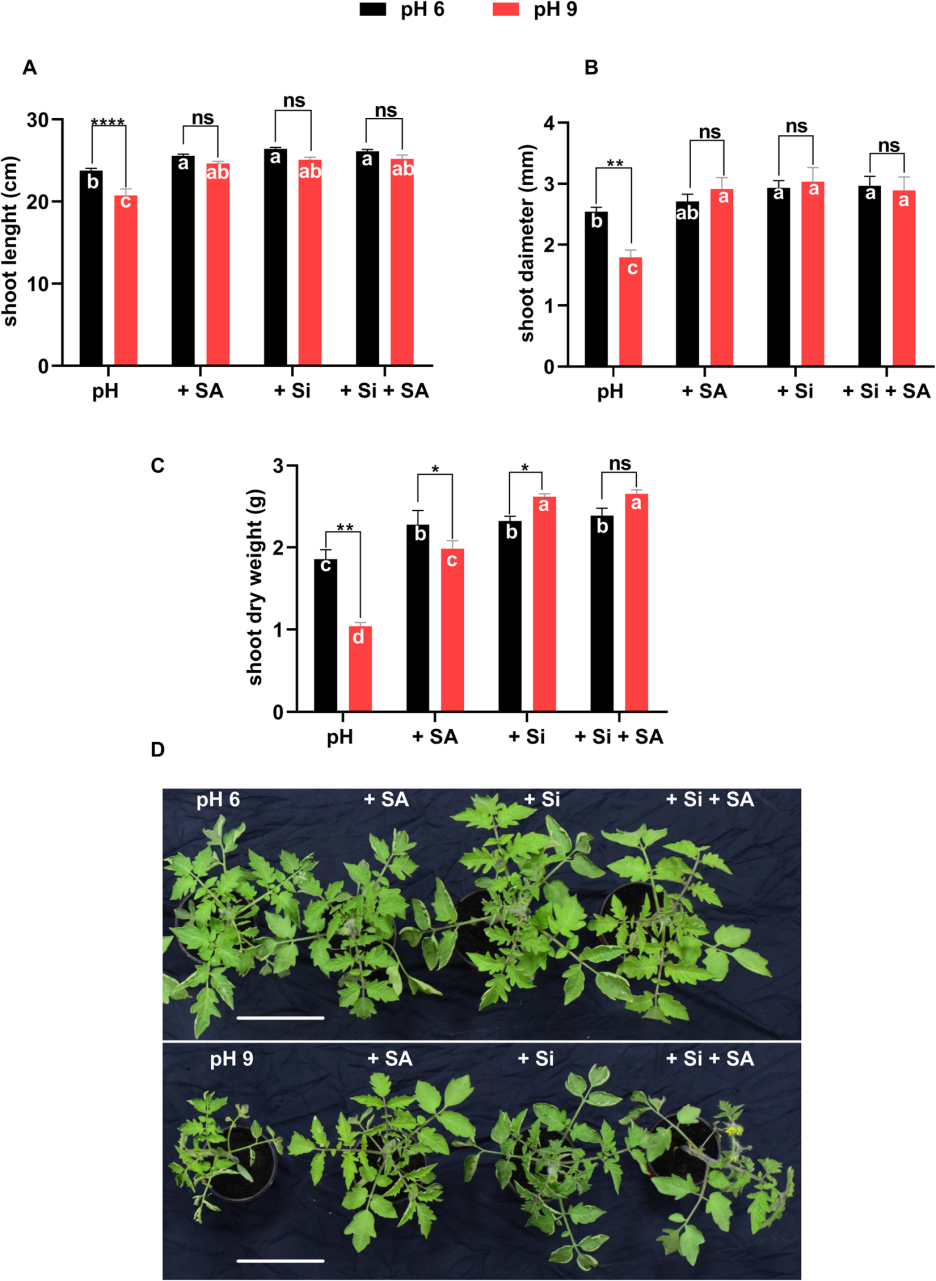
Effects of alkaline stress on tomato plants. (**A**) Shoot length. (**B**) Shoot diameter (**C**). (**D**) Photograph of the shoot, illustrating the typical differences among all treatments at each pH. Tomato seedlings were grown in pots. A solution at pH 6.0 and pH 9.0, respectively, was applied daily to each pot. *,**, *** and **** indicate a significant difference between treatments at a given pH where *P* < 0.05, 0.01 and 0.001, respectively, and “ns” indicates non-significant differences. Different letter (s) indicate a significant difference (*P* < 0.05) among all treatments by DMRT test.

The results showed that the shoot length, diameter, fresh (FM) and dry masses (DM) were significantly lowered by high-pH compared with pH 6.0. No significant differences were observed in shoot growth among Si, SA and Si + SA treatments at each pH, respectively (Fig. 1A–C and Supplementary Fig. S1). However, shoot DM was significantly higher in Si-treated plants at pH 9.0 than at pH 6.0. A similar prospect was also shown by SA treatment, where the shoot DM was significantly higher at pH 6.0 than at pH 9.0 (Fig. 1D). In conclusion, alkalinity has hindered the shoot length as compared to control, whereas the SA and Si treatments have significantly improved the shoot diameter and DM during alkaline stress. In comparison, the effect of SA and Si was not significantly different from each other. Thus, results support that exogenously applied sole or combined Si/SA improves plant growth under alkaline conditions.

**Figure 2:**
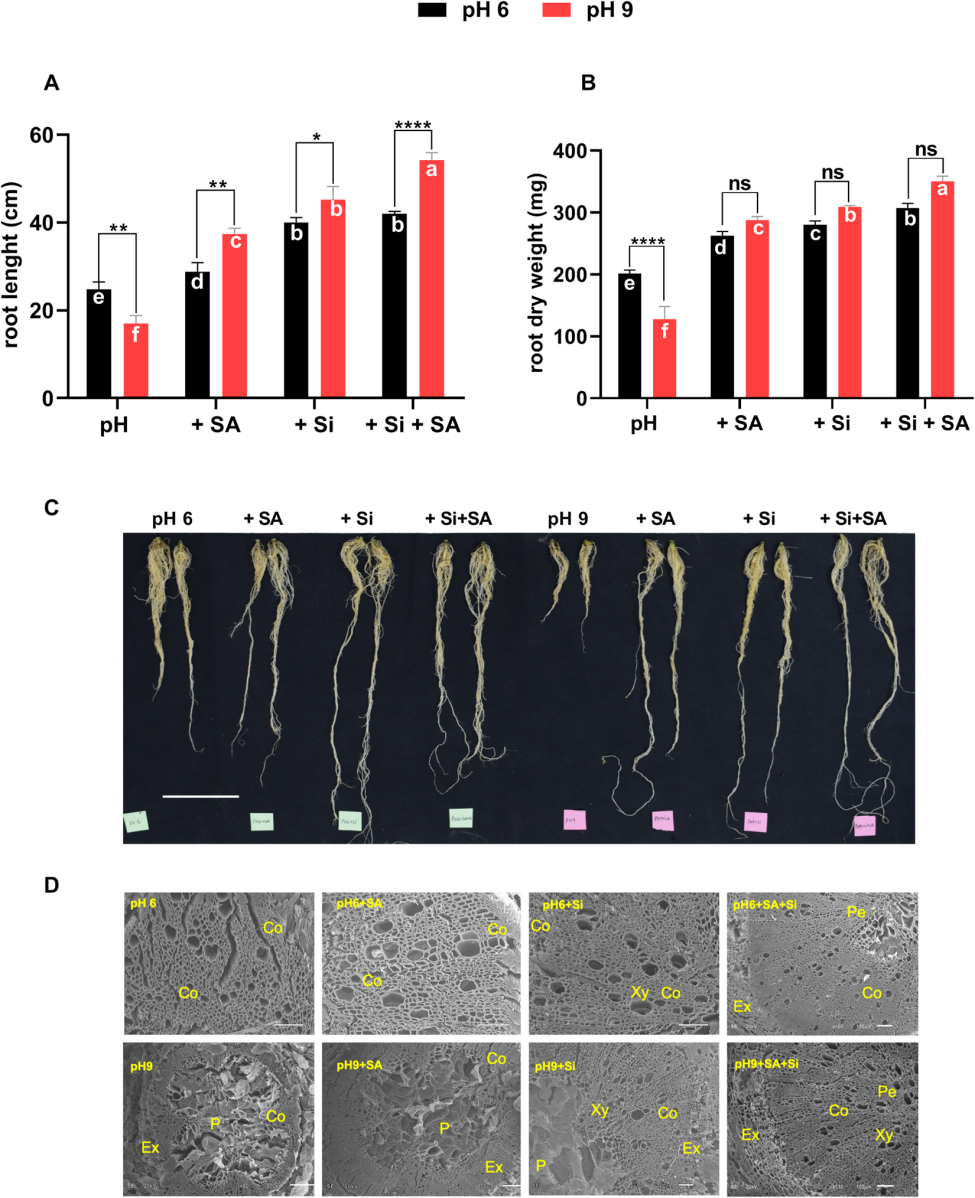
Effects of alkaline stress on (**A**) root length and (**B**) root dry weight. (**C**) Photograph of the root, illustrating the typical differences between all treatments at each pH. (**D**) Scanning electron micrographs of the root in response to pH, and Si and SA application. Co-cortex, p-pericycle, Ex-exodermis, Xy-xylem. A solution at pH 6.0 and pH 9.0, respectively, was applied to each pot 3–4 times a week. *,**, *** and **** indicate a significant difference between treatments at a given pH where *P* < 0.05, 0.01 and 0.001, respectively, and “ns” indicates non-significant differences. Different letter (s) indicate a significant difference (*P* < 0.05) among all treatments by DMRT test.

Root morphological analysis revealed significantly higher root length in plants exposed to pH 6.0 than pH 9.0 (Fig. 2A). Interestingly, exogenously applied sole Si, SA and combined Si + SA significantly increased the root length at pH 9.0 relative to the corresponding treatments at pH 6.0. Si and Si + SA treatments showed a higher root length at pH 6.0 than at pH 9.0 (Fig. 2A). Root’s fresh and dry biomass were significantly higher at pH 6.0 than at pH 9.0. However, during Si, SA and Si + SA treatments at both kinds of pH, the root’s FM and DM were significantly higher. This was more pronounced in pH 9.0 than in pH 6.0, whereas, among different treatments, a combine treatment of Si + SA showed potent function in increasing root growth than sole treatments (Fig. 2B,C and Supplementary Fig. S1). Figure 2D illustrates the typical differences in the root morphology among all treatments at each pH. These effects were also evidenced by SEM analysis of roots treated with Si/SA under pH stress. In comparison to pH 6.0, the results showed more intact internal root structure with less damages to the cortex, exodermis and pericycle regions after Si and SA applications. Contrarily, pH 9.0 exponentially devastated the internal root architecture by damaging the pith and pericycle regions, whereas the root development reduced shrinkages in area and structure. These adverse impacts were relieved to some extent by exogenously applied Si and SA. For the Si + SA application, the root structures were more intact as compared to exogenously applied Si and SA, separately, during pH 9.0 stress (Fig. 2D).

### Estimates of chlorophyll contents (*a* and *b*), RWC and antioxidant enzymes

**Figure 3:**
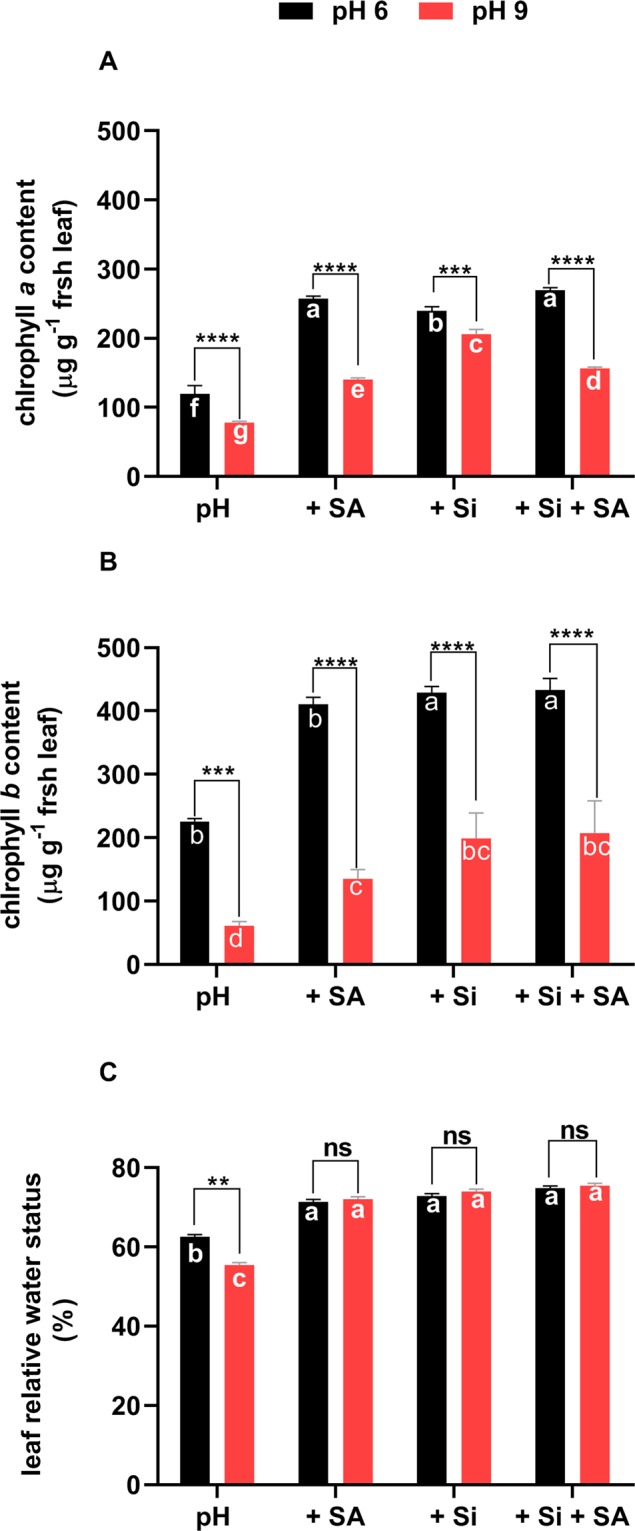
Estimates of the (**A**) chlorophyll *a*, (**B**) chlorophyll *b* and (**C**) leaf relative water content in response to solution pH. *,**, *** and **** indicate a significant difference between treatments at a given pH where *P* < 0.05, 0.01 and 0.001, respectively, and “ns” indicates non-significant differences. Different letter (s) indicate a significant difference (*P* < 0.05) among all treatments by DMRT test.

To explore the physiological and biochemical mechanisms by that plants exhibited improved root growth under alkaline conditions, we compared the effects of control and alkaline stress on Chl *a* and *b*, RWC and the antioxidant defence enzymes of the tomato plants under control (pH 6.0) and alkaline conditions (pH 9.0) and with application of Si and SA. Both Chl *a* and Chl *b* were significantly lowered by high-pH. Sole Si, SA and combined Si + SA treatments significantly increased chlorophyll contents at each pH, except for the SA treatment at pH 9.0, that was not significantly different from control pH 9.0 (Fig. 3A,B). Leaf water contents were also higher in sole SA/Si and combined Si + SA treatments as compared with the control at each pH (Fig. 3C). It suggests that under alkaline condition, Si, SA and Si + SA treatments improved the chlorophyll contents, and in turn, the photosynthetic process and plant growth, to mitigate the detrimental effects of high-pH.

Shoot and root antioxidant defence enzymes (APX, CAT, POD and PPO) activities, after 5 weeks of treatments, are shown in Table 1. POD activity in tomato shoot and root was significantly higher in SA, Si and Si + SA treatments at each pH as compared with the corresponding controls. No significant differences in POD activity was observed between the treatments. At pH 9.0, the control showed non-significant difference with treatments (Table 1). APX, CAT and PPO activity levels in tomato shoot were significantly higher at pH 9.0 than at pH 6.0, irrespective of the treatment, except for the CAT activity in response to Si application (Table 1). Similarly, all the three antioxidant enzymes were significantly higher in SA, Si and Si + SA treatments at each pH as compared with the control at each pH (Table 1). No significant differences were observed in APX, CAT and PPO concentrations in tomato root exposed to pH 9.0 treatments as compared with the same treatments at pH 6.0, except for pH 9.0 alone versus pH 6.0 alone. Besides, significantly higher APX was observed in Si application at pH 9.0 as compared with the same treatment at pH 6.0 (Table 1). These results imply that Si, SA and Si + SA treatments, improved the antioxidant defence system and this could be one of the reasons for enhanced plant growth under alkaline conditions. Overall, a combined application of Si + SA have been proposed to activate oxidative stress defence mechanisms as compared to SA/Si alone treatments during pH 9.0 stress.

**Table 1:** Effect of exogenously applied Si, SA and their combination on root and shoot’s malonaldehyde (MDA), the superoxide radical (•O_2_^–^) level and the antioxidant defence enzymes, such as ascorbate peroxidase, catalase, peroxidase and polyphenol peroxidase under normal and alkaline stress conditions. Different letter (s) in each row indicate a significant difference (*P* < 0.05) among all treatments by DMRT.

		pH 6				pH 9			
Parameters	Organ	Control	+SA	+Si	+Si + SA	Control	+SA	+Si	+Si + SA
MDA (nmol mg^−1^ FW)	Shoot	1.30 ± 0.03^b^	1.02 ± 0.05^c^	1.03 ± 0.04^c^	0.94 ± 0.17^c^	1.78 ± 0.01^a^	1.53 ± 0.04^b^	1.42 ± 0.04^b^	1.20 ± 0.04^c^
	Root	2.50 ± 0.34^c^	2.62 ± 0.44^c^	1.99 ± 0.33^cd^	2.70 ± 0.26^c^	4.67 ± 1.00^a^	3.96 ± 0.81^b^	2.29 ± 0.10^c^	3.06 ± 0.67^c^
O_2_^−^ (μmol mg^−1^ FW)	Shoot	19 ± 0.20^c^	22.58 ± 0.84^b^	19.75 ± 0.20^c^	18.91 ± 0.42^c^	29.41 ± 1.19^a^	22.08 ± 0.62^b^	17.58 ± 0.23^d^	17.33 ± 0.11^d^
	Root	12.75 ± 0.70^c^	14.25 ± 0.31^b^	11.25 ± 0.11^d^	11.75 ± 0.31^d^	16.5 ± 1.32^a^	13.25 ± 0.84^c^	11.5 ± 0.20^d^	11.75 ± 0.11^d^
POD (μmol mg^−1^ protein)	Shoot	0.84 ± 0.04^bc^	1.34 ± 0.03^a^	1.31 ± 0.05^a^	1.41 ± 0.01^a^	1.01 ± 0.02^b^	1.28 ± 0.01^a^	1.27 ± 0.01^a^	1.30 ± 0.03^a^
	Root	0.57 ± 0.08^ab^	0.65 ± 0.01^a^	0.76 ± 0.01^a^	0.76 ± 0.01^a^	0.66 ± 0.03^a^	0.69 ± 0.01^a^	0.73 ± 0.02^a^	0.70 ± 0.01^a^
PPO (μmol mg^−1^ protein)	Shoot	1.30 ± 0.03^c^	1.69 ± 0.02^bc^	1.84 ± 0.03^b^	2.01 ± 0.01^b^	1.73 ± 0.01^bc^	1.95 ± 0.02^b^	2.30 ± 0.02^a^	2.53 ± 0.03^a^
	Root	0.73 ± 0.02^b^	0.89 ± 0.01^a^	0.98 ± 0.02^a^	0.99 ± 0.04^a^	0.88 ± 0.01^a^	0.90 ± 0.01^a^	0.95 ± 0.04^a^	0.98 ± 0.01^a^
APX (μmol mg^−1^ protein)	Shoot	0.74 ± 0.02^cd^	1.33 ± 0.08^b^	1.17 ± 0.03^bc^	1.55 ± 0.03^a^	0.90 ± 0.02^c^	1.07 ± 0.02^bc^	1.09 ± 0.01^bc^	1.21 ± 0.08^b^
	Root	0.34 ± 0.009^b^	0.44 ± 0.03^a^	0.038 ± 0.05^a^	0.42 ± 0.02^a^	0.42 ± 0.01^a^	0.43 ± 0.01^a^	0.42 ± 0.02^a^	0.44 ± 0.02^a^
CAT (μmol mg^−1^ protein)	Shoot	3.35 ± 0.94^g^	21.3 4 ± 0.67^e^	53.02 ± 6.36^b^	33.63 ± 3.09^d^	11.97 ± 7.12^f^	40.66 ± 1.62^c^	43.38 ± 2.18^c^	74.76 ± 3.34^a^
	Root	14.63 ± 0.75^d^	18.46 ± 9.14^d^	24.70 ± 6.02^d^	38.98 ± 12.82^b^	32.77 ± 2.05^c^	33.69 ± 8.33^c^	41.89 ± 7.30^b^	51.57 ± 8.10^a^

### Lipid peroxidation and •O_2_^−^ measurements from tomato root and shoot

Shoot lipid peroxidation levels were significantly higher in pH 9.0 treatments as compared with pH 6.0. However, Si and Si + SA treatments significantly reduced lipid peroxidation levels at both pH 6.0 and 9.0, although this decrease was more noticeable at pH 9.0 (Table 1). Surprisingly, higher lipid peroxidation levels were detected in the tomato root than the shoot. Like the shoot results, the lipid peroxidation levels were significantly higher at high-pH treatments as compared with the same treatments at pH 6.0. However, Si and Si + SA treatments have significantly lowered the lipid peroxidation levels as compared with alone pH 9.0 (Table 1). No significant differences were observed in root lipid peroxidation levels among all treatments at pH 6.0 (Table 1). Both root and shoot had significantly higher •O_2_^−^ levels in pH 9.0 treatments as compared with the pH 6.0. Contrary to this, Si, SA and Si + SA treatments significantly reduced the •O_2_^−^ levels under high-pH conditions, suggesting that Si/SA have significantly regulated alkalinity induced oxidative stress through increased antioxidant enzyme activities.

### Effects of Si and SA on the Na^+^ toxicity and K^+^ homeostasis

Tomato plants that are suffering from alkaline stress have to cope with excessive Na^+^ in the growth medium, so we compared its effect on the Na^+^ and K^+^ concentrations in the shoot and root parts. While there were no significant differences in the tomato shoot Na^+^ concentrations between the treatments at each pH levels. The results showed a significant reduction in Na^+^ in plants treated with SA under alkaline conditions as compared with pH 9.0 alone (Table 2). Only in combined Si + SA treatment a significant difference in the root Na^+^ concentration was observed, that was higher at pH 9.0 than at pH 6.0. Exposure to alkaline conditions significantly lowered shoot and root K^+^ concentrations. However, Si, SA and Si + SA treatments significantly increased shoot and root K^+^ concentrations in plants under alkaline treatment (Table 2). Overall, Si and combined Si + SA has more significant effects on ion homeostasis than sole SA treatment.

**Table 2:** Effects of alkaline stress on the concentrations of Si, Na^+^ and K^+^ in the shoot and root of tomato plants grown under normal and alkaline stress conditions with/without exogenously applied Si, SA and Si + SA. Different letter (s) in each row indicate a significant difference (*P* < 0.05) among all treatments by DMRT.

Parameters	Organ		pH 6				pH 9		
		Control	+SA	+Si	+Si + SA	Control	+SA	+Si	+Si + SA
Silicon (µmol g^−1^)	Shoot	417.77 ± 77.22^f^	447.72 ± 20.38^e^	986.7 ± 51.043^b^	1071 ± 105.34^a^	576.43 ± 74.34^d^	359.72 ± 24.21^g^	852.41 ± 66.16^c^	841.2 ± 50.18^c^
	Root	152.58 ± 12.78^e^	114.16 ± 29.39^f^	556.47 ± 42.55^a^	518.15 ± 19.11^d^	157.11 ± 9.12^e^	100.85 ± 9.61^f^	630.08 ± 50.42^a^	550.75 ± 42.55^c^
Sodium (µmol g^−1^)	Shoot	195.33 ± 34.83^b^	142.01 ± 8.04^e^	210.00 ± 16.33^a^	198.01 ± 9.89^b^	178.33 ± 20.54^c^	157.65 ± 6.12^d^	174.96 ± 18.78^c^	159.59 ± 16.73^d^
	Root	2205.5 ± 156.42^b^	2055.01 ± 69.81^d^	2149.5 ± 81.24^c^	2339.1 ± 162.48^b^	2250.9 ± 285.78^b^	1735.9 ± 257.90^e^	2153.7 ± 155.66^c^	2799.2 ± 327.42^b^
Potassium (µmol g^−1^)	Shoot	5720.8 ± 128.83^f^	6611.1 ± 134.53^e^	10532.0 ± 1191^b^	13525.0 ± 1471^a^	3920.3 ± 139.07^g^	5621.2 ± 473.55^f^	10268 ± 426.8^c^	8262 ± 668.51^b^
	Root	2655.1 ± 249.1^g^	4509.3 ± 84.01^d^	4758.8 ± 154.0^c^	4352.3 ± 168.19^e^	3394.5 ± 201.0^f^	4934.1 ± 150.77^b^	5057.3 ± 76.71^a^	5100.3 ± 242.85^a^

### Effects of Si and SA on the endogenous SA and ABA levels

**Figure 4:**
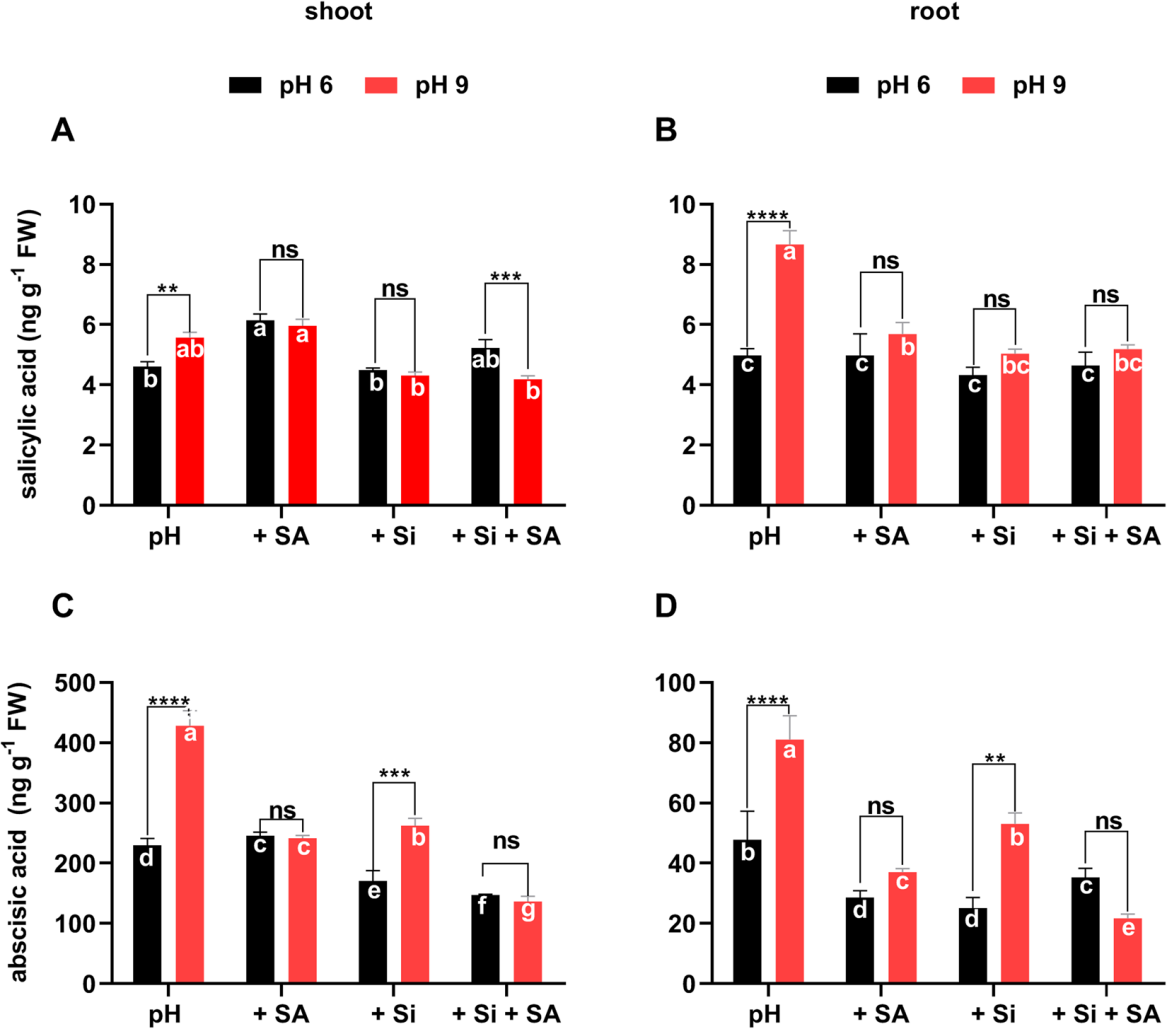
Measurements of the endogenous shoot and root (**A**,**B**) salicylic acid and (**C**,**D**) abscisic acid levels in tomato plants grown under pH 6.0 and alkaline stress conditions with/without exogenously applied Si, SA and Si + SA. *,**, *** and **** indicate a significant difference between treatments at a given pH where *P* < 0.05, 0.01 and 0.001, respectively, and “ns” indicates non-significant differences. Different letter (s) indicate a significant difference (*P* < 0.05) among all treatments by DMRT test.

To explore the cross-talk between exogenous Si and SA with endogenous the SA and ABA under alkaline conditions, we measured the endogenous SA and ABA levels in both shoot and root of tomato seedlings. In alkaline conditions, high endogenous SA levels were observed at high pH alone and SA treatment, but the SA level was significantly lowered by exogenously applied Si alone and Si + SA (Fig. 4A). No significant differences were observed among treatments as considering each pH separately. However, significantly higher endogenous SA levels were observed at pH 9.0 than at pH 6.0. In addition, Si + SA treatment showed significantly different SA levels at each pH (Fig. 4A). In the root, only SA levels, that were higher at pH 9.0 than pH 6.0, were significantly different (Fig. 4B). Similar to the shoot data, Si alone and Si + SA significantly reduced the endogenous SA levels at high pH as compared with the high-pH control (Fig. 4B).

Exposure to alkaline conditions has led to significantly higher endogenous ABA levels in tomato shoot (Fig. 4C), but this effect was significantly alleviated by Si, SA and Si + SA treatments. In the comparison, only at pH 9.0 alone and Si treatment showed a significant difference in the ABA levels, that were higher than the corresponding treatments at pH 6.0 (Fig. 4D). Similar to the shoot results, higher ABA levels were found in the root exposed to alkaline conditions, that were significantly lowered by Si, SA and Si + SA applications. In the comparison between pH treatments, pH 9.0 alone and Si treatment showed significantly higher ABA levels, whilst Si + SA treatment showed significantly lowered ABA levels under the alkaline conditions as compared with the same treatments at pH 6.0. In cross-talk of endogenous ABA/SA, the concentrations of ABA were significantly higher than SA in shoot parts as compared with root during different pH treatments.

### Expression of *LSi1* genes involved in endogenous SA metabolism and plasma membrane H^+^-ATPase genes in tomato exposed to high-pH treatments

**Figure 5:**
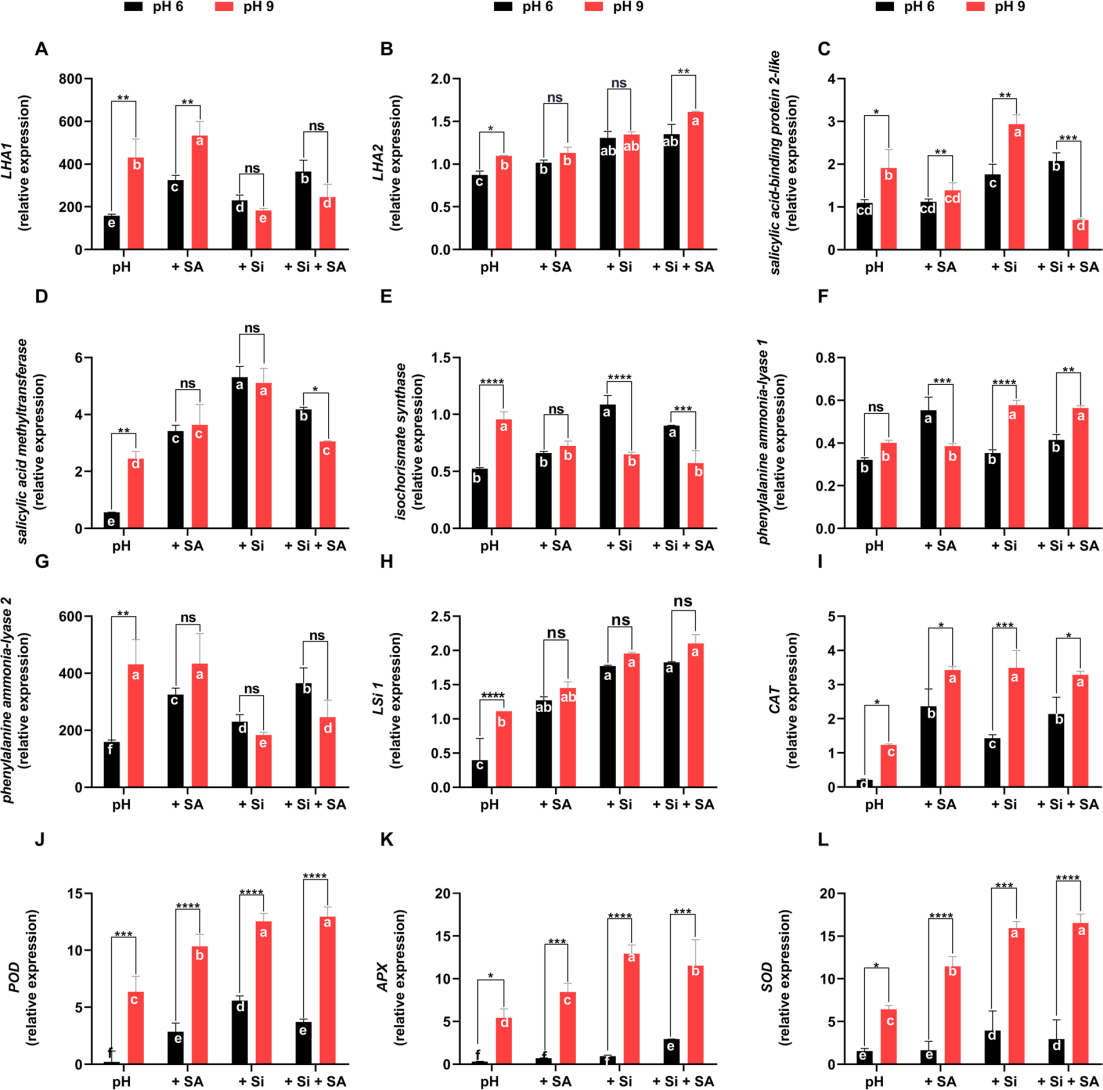
Effects of alkaline stress on the expression of related genes in tomato plants. (**A**) *LHA1*, (**B**) *LHA2*, (**C**) *SA-binding protein-like 2*, (**D**) *SA methyltransferase*, (**E**) *isochorismate synthase*, (**F**) *PAL-1*, (**G**) *PAL-2*, (**H**) *LSi1*, (**I**) *catalase*, (**J**) *peroxidase*, (**K**) *ascorbate peroxidase* and (**L**) *superoxide dismutases*. Total RNA was extracted from tomato plants grown under pH 6.0 and alkaline stress conditions (pH 9.0) with/without exogenously applied Si, SA and Si + SA for 5 weeks. Transcript levels were measured by real-time qPCR. *Actin* was used as an internal control. Error bars are calculated based on three biological replicates. *,**, *** and **** indicate a significant difference between treatments at a given pH where *P* < 0.05, 0.01 and 0.001, respectively, and “ns” indicates non-significant differences. Different letter (s) indicate a significant difference (*P* < 0.05) among all treatments by DMRT test.

The plasma membrane H^+^-ATPase plays an essential role in plants by modulating proton secretions into the rhizosphere. Hence, we measured the expression levels of the plasma membrane H^+^-ATPase genes *LHA1* and *LHA2* in tomato plant after 5 weeks of growth at pH 6.0 and pH 9.0 under otherwise the same conditions. Furthermore, we also studied the genes involved in Si uptake (*LSi-1*), SA biosynthesis (*ICS*, *SAMT1*, *SABP2*, *SAMT*, *SABP2*, *ICS*, *PAL1* and *PAL2*) and the antioxidant defence system (*CAT*, *POD*, *APX* and *SOD*). Since, H^+^ and Si uptake is broadly regulated in root part therefore, the responsible *LHA1, LHA2* and *LSi-1* genes were analysed in root part only. We tried to elucidated the gene expression in shoot part, however, either low or no expression pattern was revealed in shoot part. *LHA1* and *LHA2* were significantly up-regulated at pH 9.0 compared with pH 6.0. Si application did not change the expression levels of both genes at each pH. SA application significantly up-regulated *LHA1* expression, and Si + SA up-regulated *LHA2* expression at pH 9.0 compared with pH 6.0 (Fig. 5).

In case of SA biosynthesis related genes, *SABP2* was up-regulated by Si and SA, and down-regulated by Si + SA treatment in alkaline conditions that is relative to the same treatment at pH 6.0 (Fig. 5). *SAMT1* expression was up-regulated by pH 9.0 alone and down-regulated by Si + SA treatment as compared with the same treatments at pH 6.0 (Fig. 5). *ICS* was up-regulated by pH 9.0 alone and down-regulated by Si and Si + SA treatments (Fig. 5). *PAL1* was up-regulated by Si and Si + SA treatments and down-regulated by SA, and *PAL2* was up-regulated only by pH 9.0 alone compared with pH 6.0 (Fig. 5). *APX*, *CAT*, *POD* and *SOD* genes were up-regulated by Si, SA and Si + SA treatments in plants under alkaline conditions compared with the same treatments at pH 6.0 (Fig. 5). At pH 9.0, the up-regulation of these genes was significantly higher in Si, SA and Si + SA treatments than pH 9.0 alone.

## Discussion

The current study demonstrated adverse effects on the overall plant growth of tomato plants under alkaline conditions. We observed a dramatic decline in the shoot diameter, shoot and root length, and the FM and DM of the tomato plants due to the pH 9.0 condition, possibly because of a high-pH rhizospheric environment, that caused osmotic stress by impairing ion homeostasis, in turn, hampering overall growth performance. However, the application of Si and SA at high pH ameliorated the plant growth and biomass. Consistent with our results, it has been shown that exogenous application of Si and SA counter the adverse effects of abiotic stress and restore plant growth in various crop plants^18,84,85^.

High-pH conditions dramatically decreased the leaf chlorophyll contents (Chl *a* and Chl *b*) in the present study, and similar results were described by Mir *et al*.^20^ and Abdel Latef and Tran^25^. Both studies reported significantly lowered chlorophyll contents in plants subjected to alkaline stress. Our results also confirm the finding of Li *et al*., who observed a higher chlorophyll content and photosynthetic rate in Dongdao-4 rice plants than Jigeng-88 plants due to greater tolerance to saline–alkaline stress conditions. It was further suggested that the higher chlorophyll content and photosynthetic rate might be the reason for the improved root growth in Dongdao-4 rice plants under saline-alkaline stress conditions. The reduction in chlorophyll content might be because of (i) reduced enzymatic activity of protochlorophyllide reductase and α-aminolevulinic acid dehydratase, as both are involved in chlorophyll biosynthesis^86^, and (ii) enhancement of the oxidative stress, that causes chloroplast injury^25^. In current study, Si/SA treatments have significantly improved the chlorophyll contents during alkaline stress, suggesting amelioration in plant’s essential biochemical contents.

The reduction in LRWC by alkaline stress have been caused by osmotic stress that was posed by high pH environment, that in-turn also activates the accumulation of osmoprotectant proline and causes the water deficit conditions, thus, slowing down water uptake. Exogenously applied Si and SA triggered LRWC recovery in alkaline-stressed plants to the level of the control. Likewise, Abdel Latef and Tran^25^ reported that Si application improved the RWC in maize seedlings under alkaline-stressed conditions and Hayat *et al*.^87^ found recovery in the LRWC of tomato plants by SA application in salinity-stressed conditions. The improved LRWC in tomato plants under Si and Si + SA treatments might be because of the deposition of Si as silicate crystals in epidermal tissues, as this constitutes an obstacle to water transpiration through the stomata and cuticle, helping to reduce the toxicity of alkalinity^88^.

Alkaline stress tends to also increases the ROS production^12,13,25^ and, ultimately, aggravates oxidative damage to lipids, proteins and nucleic acids. Therefore, the rapid activation of ROS-scavenging molecules is required for alkaline stress adaptation and tolerance. High levels of lipid peroxidation triggered by ROS could promote the generation of MDA, that is commonly used as a primary indicator of oxidative stress damage to the lipid bilayer under chemical stresses^91,89^. It was found that alkaline conditions significantly enhanced the •O_2_^−^ and MDA contents, but the increase was remarkable in pH 9.0-treated plants in contrast to Si- and SA-treated plants under alkaline conditions. Comparable reductions in MDA contents by Si addition have been reported in maize and rice^25,90^. The present result is also supported by Gunes *et al*.^91^, who found that the overproduction of MDA in maize under salinity stress was significantly reduced by SA application. APX, CAT, POD, PPO and SOD comprises one of the leading enzymatic system that detoxifies ROS^92^. In the present study, alkaline stress lowered the APX, CAT, POD and PPO contents, but Si and SA application mitigated this effect. These results suggest that both Si and SA enhance the antioxidant system whilst it was significant in combined Si + SA to protect plants against alkalinity-induced oxidative damage, as evidenced by the decreased contents of •O_2_^−^ and MDA. The increase in antioxidant activities in the present study validates the findings of Eraslan *et al*.^93^. This suggests that current findings of increased antioxidant defence system by Si/SA treatment in tomato seedlings was involved in counteracting negative impacts of alkaline stress.

Further to coping ROS generation due to alkalinity, plant roots also absorb several minerals, including essential minerals (such as K^+^) and some other minerals (such as Na^+^), that are toxic for normal plant growth and development^94^. Growth inhibition is one of the most common effects of excessive Na^+^ concentration in plants and might have been correlated with the K^+^ deficiency. Hence, the mineral balance in plants is essential for physiological functions. For example, the K^+^/Na^+^ concentrations ratio and homeostasis usually regulate plant growth rate. Either high Na^+^ or low K^+^ in the soil signifies a stress condition that can severely affect plant performance and agricultural productivity^94^. We used sodium silicate as the exogenous Si source in this experiment. Hence, to ensure that excessive Na^+^ was not the main cause of stress, we measured the Na^+^ concentrations in root and shoot. We did not observe any difference in the Na^+^ concentration between plants treated with and without sodium silicate (Si), that suggests that the toxicity was not because of high Na^+^ and was mainly because of high pH. However, we observed high-K^+^ concentrations in both root and shoot of plants treated, respectively, with Si, SA and Si + SA, suggesting that the tolerance of the tomato plants under alkaline conditions was also achieved by the increased K^+^ level associated with the exogenously applied Si and SA. Besides, the Si uptake was confirmed by measuring the Si concentrations in both shoot and root.

In addition to Si, SA also has a role in plant adaptation to environmental stimuli, such as salinity, drought and low temperature^95,96^. However, to our knowledge, there are no prior reports on the interactive effects of SA and Si on the physiological and biochemical responses of plants under alkaline stress. Although poorly understood, an essential role for SA in plant adaptation to alkaline stress is supported by the current study. We observed that the high endogenous SA levels in both root and shoot, induced under alkaline conditions, were alleviated by treatment with Si or Si + SA. It suggests that the endogenous SA concentration in those plants was not critical because the exogenously applied Si and SA mitigated the alkaline stress, as evidenced by the improved plant growth and lowered MDA levels under these conditions. Furthermore, the exogenous SA regulates the ROS generation and activating antioxidant defence mechanism to combat stress conditions, however, lower levels of endogenous SA at pH 9.0 also reveals that considerably reduced amount of stress was experienced by the tomato seedlings. SA signalling has often been involved with either antagonistic and synergistic responses with other endogenous phytohormones such as ABA^30,93^.

ABA is a critical plant hormone, with an important role during different phases of the plant life cycle, including seed dormancy, seed and leaf development and plant responses to various environmental stresses^97–99^. The ABA content increases in plants exposed to salinity stress^100,101^, resulting in diminished stomatal conductance^102^, and ultimately, affecting chlorophyll synthesis and photosynthesis. In our results, the ABA levels were significantly lowered by Si, SA and Si + SA treatment in plants under alkaline conditions. In agreement with this result, Kim *et al*.^28^ found lower ABA levels in plants treated with Si under heavy-metal stress conditions. The same authors further suggested that ABA has an antagonistic behaviour with SA biosynthesis in Si-treated plants, and also speculates an essential role of ABA in Si-mediated tolerance in plants in response to environmental stress. A lower ABA content in plant has also been coined for the perception of reduced levels of stress factor^28,61^, hence, in current case both Si and SA have counteracted the high pH whilst extending tolerance by continually increase the plant growth attributes. It might be correlated with increase in Si content to help the plant to activate less ABA, whereas, the responses of SA could be activated as was revealed by gene expression of SA receptor and biosynthesis related genes during Si + SA applications.

To further explore the molecular mechanisms behind the improved growth in tomato plants under alkaline conditions in response to Si and SA applications, we assessed the gene expression patterns involved in Si uptake, SA biosynthesis, the antioxidant defence system and the acidification of the rhizosphere. *Phenylalanine ammonia-lyase* (*PAL*) catalyses the first step of the phenylpropanoid pathway, that produces precursors for many critical secondary metabolites, including SA^103^. A decrease in *PAL* activity decreases the SA levels^104^. This is correlative to our current findings where we found lower activation of SA in pH stress furing Si/SA application. SA is also synthesised *via* the isochorismate pathway^105^. Wildermuth, *et al*.^105^ identified the *ICS1* gene in the *Arabidopsis* genome. The same authors further noted that *ICS1* expression correlated with SA accumulation and expression of the *SA-associated PR-1* gene. *Salicylic acid methyltransferase* (*SAMT1*) has an essential role in methylated SA (MeSA) biosynthesis, and *SA-binding protein 2* (*SABP2*) hydrolyses MeSA to SA^106,107^. Therefore, we measured the expression levels of *ICS*, *PAL1*, *PAL2*, *SABP2* and *SAMT* to elucidate the involvement of SA in extending tolerance to tomato seedlings during alkalinity alike with other abiotic stress conditions. SA has been inter-correlated with tolerance to crops via antagonizing with ROS burst in abiotic stresses^40,96^. In correlation with biochemical data of antioxidant enzymes, we also assessed the expression of the candidate genes involved in antioxidant defence systems, such as *APX*, *CAT*, *POD* and *SOD*. The results showed increase in mRNA gene expression of these genes that corroborates with previous findings^108–110^. This was also revealed that Si in combination with SA have further enhanced the defensive mechanisms however, the relative expression of gene encoding antioxidant enzymes has been least understood in alkalinity.

Similarly, the plasma membrane H^+^-ATPase is critical in plant adaptation to alkaline conditions, as it mediates proton secretion^2,8,9^. *LHA1* and *LHA2* are the crucial members of the H^+^-ATPase in tomato. Our results showed that tomato H^+^-ATPase were significantly up-regulated as relative expression of both *LHA1* and *LHA2* were significantly higher in Si and SA application. It infers that high-pH enhanced the H^+^-ATPase activity, and so more protons were secreted into the rhizosphere, that has decreased the rhizospheric pH, ultimately enhancing plant’s stress tolerance^111^–^113^. A higher Si uptake then tends to increase rigidity to cellular masses helped in translocation of ionic imbalance too – correlating with H^+^-ATPase related expression. Si uptake was further validated by the expression pattern of *SlLsi1* and *SlLsi2* genes. The function of *SlLsi1* and *SlLsi2* is important during Si transport as revealed by *Lsi* knockout that markedly decreased Si uptake, however, it is poorly understood in alkaline stress conditions^114^. Si accumulation in plants is attributed to an effective uptake system facilitated by both *Lsi1* and *Lsi2*^115^. Previously, Ma, *et al*.^116^ has explained the role of *Oryza Sativa* (*OsLsi1* and *OsLsi2*) in Si influx and efflux, present in the plasma membrane of rice plant cells^116^. Interestingly, our results revealed that *Lsi1* genes were up-regulated by the application of Si, SA and their combination under both normal pH 6.0 and conditions. Furthermore, *Lsi1* also up-regulated under alkaline pH 9.0 as compared to normal pH 6.0, suggesting improved uptake and reducing the negative impact of ionic imbalance in soil during alkalinity. This might be due to the accumulation of Si in shoot and root, providing additional strength to root, leaf and stem structure to improve tolerance level in tomato. Previously, the role of *OsLsi1* and *OsLsi2* in heavy metal tolerance was reported by Kim, *et al*.^117^ To conclude, the results suggest that the higher expression levels of these genes in Si- and SA-treated plants might allow tomato plants to mitigate high-pH toxicity in alkaline conditions, thus contributing to their improved plant growth when exposed to alkaline conditions.

**Figure 6:**
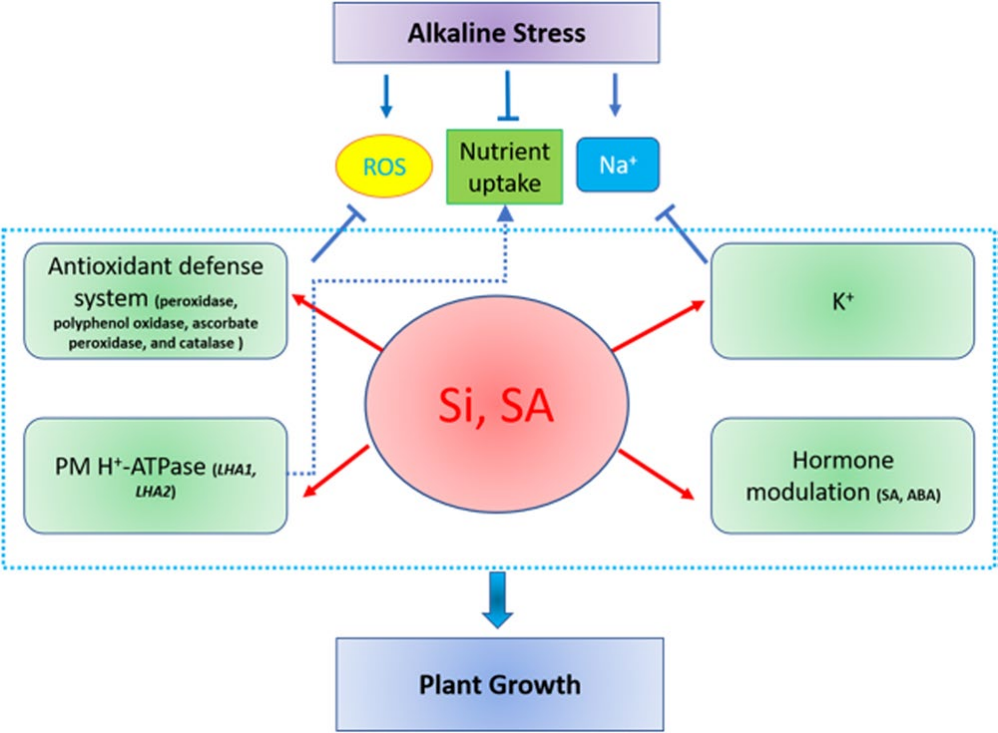
Diagram of the hypothesis of how exogenously applied Si and SA alleviate alkaline stress tolerance in tomato plants. Alkaline stress reduces plant growth by enhancing ROS, reducing nutrients uptake due to high-pH and aggravating Na^+^ toxicity. Exogenously applied Si and SA enhance the activation of the antioxidant defence system, modulate key hormones and increase the K^+^ concentration, that eventually helps the tomato plants to survive alkaline stress. Furthermore, the plasma membrane H^+^-ATPase (as per the high expression of *LHA1* and *LHA2*) pumps out H^+^ to the rhizosphere, reducing the rhizosphere pH and enhancing the nutrients uptake.

In conclusion, our results suggest that exogenously applied Si and SA confers successful tolerance of tomato plants to alkaline conditions. Firstly, both SA and Si enhance the photosynthetic potential by increasing the Chl *a* and Chl *b* contents. Secondly, both maintain water balance and shield cells from oxidative bursting. Thirdly, the duo reduce oxidative injury by regulating the antioxidant defence system and modulate the endogenous SA levels. Fourthly, both modulate the endogenous SA and ABA levels for improved plant performance during alkaline stress. Furthermore, both enhance the K^+^ concentrations in root and shoot, resulting in a low-Na^+^ ratio and high-K^+^ ratio. qPCR analysis further confirmed the critical role of Si and SA in alkaline stress tolerance of tomato by up-regulating the candidate genes. However, among treatments, the combined Si + SA showed more better prospects as compared to sole treatments. Collectively, our results suggest that exogenously applied Si and SA mitigate alkaline stress in tomato plant by enhancing the antioxidant defence system and SA modulation, and by stimulating the expression of the critical genes involved (Fig. 6). These findings will be valuable for further understanding the physiological and molecular mechanisms associated with the alkaline soil tolerance in plants.

## Supplementary information

Supplementary information
