# Supplementary material for: Silicon and salicylic acid confer high-pH stress tolerance in tomato seedlings
Source: Sci Rep. 2019 Dec 24;9:19788. doi: 10.1038/s41598-019-55651-4 (PMC6930214; doi:10.1038/s41598-019-55651-4)

# **Silicon and salicylic acid confer high-pH stress tolerance in tomato seedlings**

Adil Khan^1§^ Muhammad Kamran,^2,3§^, Muhammad Imran^4^, Ahmed Al-Harrasi^1,*^ Ahmed Al-Rawahi^1^, Issa Al-Amri^1^, In-Jung Lee^4^*, Abdul Latif Khan^1^*

**Supplementary Table S1.** Quantitative real-time PCR primer information used in the current study.

**Supplementary Table S2.** Quantitative methods and conditions for SA and ABA analysis

**Supplementary Figure S1.** Effects of alkaline stress on tomato plants. (**a**) Shoot fresh weight. (**b**) Root fresh weight (**c**). *,**, *** and **** indicate a significant difference between treatments at a given pH where *P*˂0.05, 0.01 and 0.001, respectively, and “ns” indicates non-significant differences between treatments. Different letters (lowercase) indicate a significant difference (*P*<0.05) among all treatments by two-way ANOVA.

| **Gene ID** | **Sequences (5'- 3')** | **Gene function** |
| --- | --- | --- |
| *LHA1* | Forward: GTTGGGACAGACGAAGGATGAA  Reverse: CTCAGGGAAAACACCAGCAAAC | plasma membrane H+-ATPase |
| *LHA2* | Forward: GGGTTGAGGTCACTGGCTGTAG  Reverse: GGAGGATCAAAGAGGGGTAGCA | plasma membrane H+-ATPase |
| *SAMT* | Forward: TGATTTTAACGCGATTTTTCG  Reverse: AAATGAACCAGCCACACCAC | salicylic acid methyltransferase |
| *SABP* | Forward: GTGTCATGGAGCCTGGTCTTG  Reverse: CTTGTTGATCCCTGAAGCACC | salicylic acid-binding protein 2-like |
| *ICSX1* | Forward: TGCTGCCTCATGGACATACC  Reverse: TGCGAATGGGGATTTTTCTT | isochorismate synthase |
| *PAL1* | Forward: CACTTGTGAATGGCACAGCA  Reverse: TCCGTTCATCACTTCAGCAAA | phenylalanine ammonia-lyase 1 |
| *PAL2* | Forward: AGGAAGCCGGTGGTTAGGTT  Reverse: CCGCCTCTGATAGTTGCACA | phenylalanine ammonia-lyase 1 |
| *LSI1* | Forward: TGTTCATCACCTCTGCTGTCG  Reverse: GCCCCGCCAAAATAGAAG | silicon efflux transporter LSI1 |
| *SlCAT* | Forward: GTCGATTGGTGTTGAACAGG  Reverse: AGGACGACAAGGATCAAACC | Catalase |
| *SlPOD* | Forward: TTAGGGAGCAGTTTCCCACT  Reverse: AGGGTGAAAGGGAACATCAG | cytosolic ascorbate peroxidase |
| *SlAPX* | Forward: GACTCTTGGAGCCCATTAGG  Reverse: AGGGTGAAAGGGAACATCAG | cytosolic ascorbate peroxidase |
| *SlSOD* | Forward: GGCCAATCTTTGACCCTTTA  Reverse: AGTCCAGGAGCAAGTCCAGT | Superoxide dimutase |
| ***ACT*** | Forward: GGGATGGAGAAGTTTGGTGGTGG  Reverse: CTTCGACCAAGGGATGGTGTAGC | Actin |

**Supplementary Table S1.** Quantitative real-time PCR primer information used in the current study.

**Supplementary Table S2.** Quantitative methods and conditions for SA and ABA analysis

| HPLC conditions for SA analysis | |
| --- | --- |
| Equipment  Column  Wavelength  Flow rate  Solvent A, B  Gradient (A%/B%) | Shimadzu couple with RF-10AXL fluorescence detector  C18 reverse-phase (HP hypersil ODS; particle size = 5 lm; pore  size = 120-A° water)  305 – 365 nm  1.0 ml min^-1^  Solvent A: 100% MeOH, Solvent B: 100% water in 0.5% acetic acid  (30/70) 5min → (40/60) 2.5min → (60/40) 4.5min → (30/70) 5min → (30/70) 3min |

| **Gradient** | **5min** | **2.5min** | **4.5min** | **5min** | **3min** |
| --- | --- | --- | --- | --- | --- |
| Solvent A | A : 30% | A : 40% | A : 60% | A : 30% | A : 30% |
| Solvent B | B : 70% | B : 60% | B : 40% | B : 70% | B : 70% |

Solvent A = 100% MeOH; Solvent B = 100% water in 0.5% acetic acid

ABA detailed extraction procedure and analysis

The endogenous ABA content was quantified from the frozen samples by following the protocols of Qi et al. (1998) and Kamboj et al. (1999). Plant samples were extracted with 30 mL of extraction solution containing 95% isopropanol, 5% glacial acetic acid, and 100 ng of [(±)–3,5,5,7,7,7–d^6^]– ABA. The filtrate was concentrated by a rotary evaporator. The residue was dissolved in 4 mL of 1 N sodium hydroxide solution, and then washed three times with 3 mL of methylene chloride to remove lipophilic materials. The aqueous phase was brought to approximately pH 3.5 with 6 N hydrochloric acid and partitioned three times into ethyl acetate (EtOAc). EtOAc extracts were then combined and evaporated. The dried residue was dissolved in phosphate buffer (pH 8.0) and then run through a polyvinylpolypyrrolidone (PVPP) column. The phosphate buffer was adjusted to pH 3.5 with 6 N HCl and partitioned three times into EtOAc. EtOAc extracts were combined again and evaporated. The residue was dissolved in dichloromethane (CH_2_Cl_2_), and passed through a silica cartridge (Sep-Pak; Water Associates, Milford, Massachusetts, USA) which was pre-washed with 10 mL of diethyl ether: methanol (3:2, v/v) and 10 mL of dichloromethane. ABA was recovered from the cartridge by elution with 10 mL of diethyl ether (CH_3_-CH_2_)_2_O: methanol (MeOH) (3:2, v/v). The extracts were dried and methylated by adding diazomethane for GC/MS-SIM (6890N network GC system, and 5973 network mass selective detector; Agilent Technologies, Palo Alto, CA, USA) analysis. For quantification, the Lab-Base (ThermoQuset, Manchester, UK) data system software was used to monitor responses to ions of m/e 162 and 190 for Me-ABA and 166 and 194 for Me-[^2^H_6_]-ABA.

**Supplementary Figure S1.** Effects of alkaline stress on tomato plants. (**a**) Shoot fresh weight. (**b**) Root fresh weight (**c**). *,**, *** and **** indicate a significant difference between treatments at a given pH where *P*˂0.05, 0.01 and 0.001, respectively, and “ns” indicates non-significant differences between treatments. Different letters (lowercase) indicate a significant difference (*P*<0.05) among all treatments by two-way ANOVA.


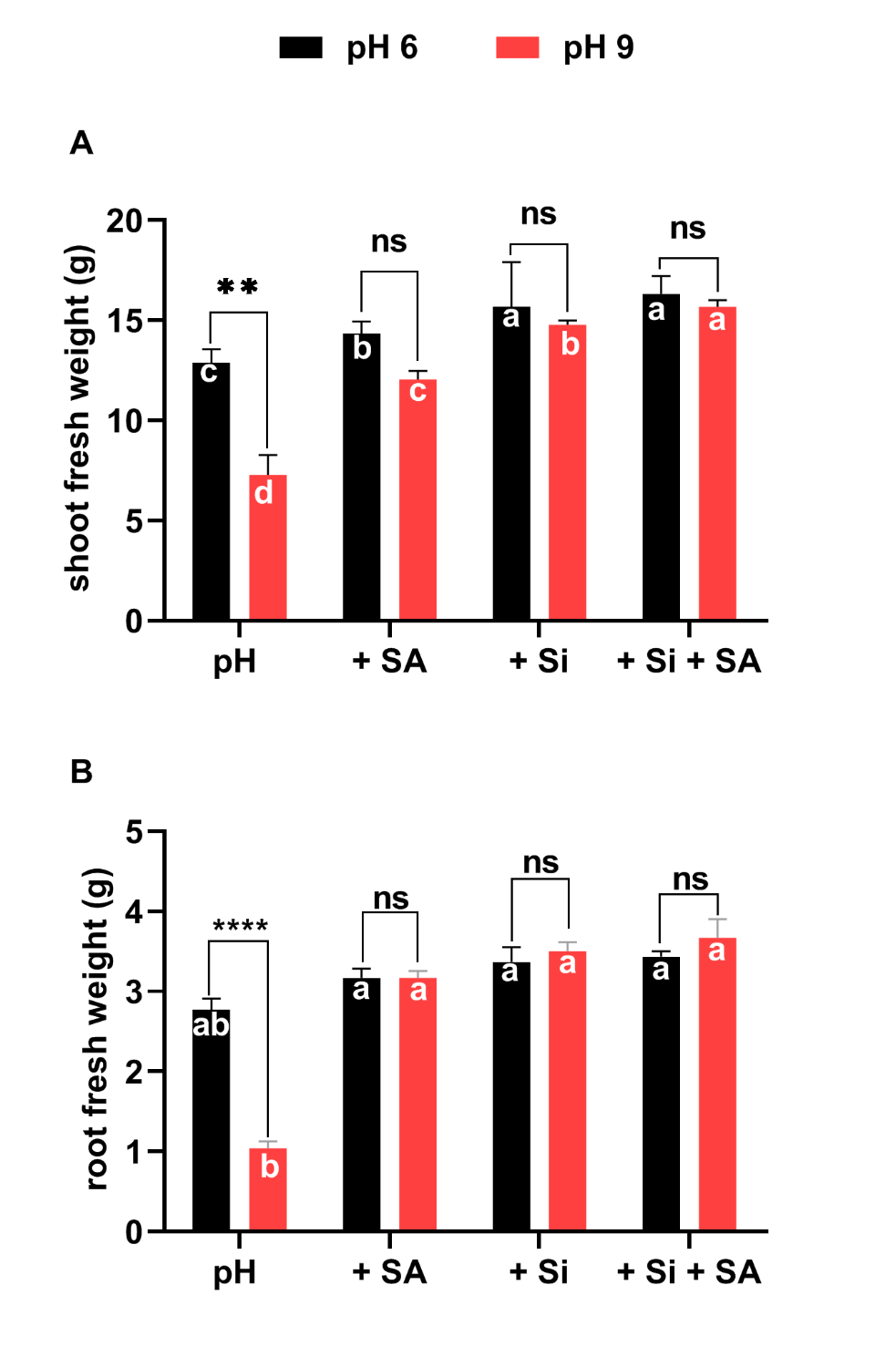

Supplement: Supplementary file 1 — Supplementary information [file 41598_2019_55651_MOESM1_ESM.docx]
